# Immediate or delayed direct restoration does not significantly influence additional endodontic treatments and 5-year tooth survival of first molars

**DOI:** 10.2340/aos.v84.44804

**Published:** 2025-10-10

**Authors:** Sara Olsson, Maria Pigg, Jesper Gustavsson, Emil Ekblom, Helena Fransson

**Affiliations:** a Department of Endodontics, Faculty of Odontology, Malmö University, Malmö, Sweden; b Dental Research Department, Public Dental Health Service, Örebro, Sweden

**Keywords:** apicoectomy, dental restoration, permanent, endodontics, tooth extraction, treatment outcome

## Abstract

**Objective:**

*In vitro* leakage studies suggest that temporary restorations only provide a short-term seal. However, it remains unclear whether the clinical outcome is impacted by the time elapsing between the completion of a root canal treatment (RCT) and the placement of a direct permanent coronal restoration. The aim was to investigate any time-dependent difference in frequencies of endodontic orthograde retreatment, apical surgery and extractions during the subsequent 5 years following root filling of first molars restored with a direct restoration depending on immediate or delayed time between completion of RCT and placement of a direct restoration.

**Material and methods:**

Data from the Swedish Social Insurance Agency was collected. In 2009, 50,314 direct restorations were registered after RCT of the first molars in individuals aged 20 years or older. The teeth were divided into five time intervals depending on time from completion of root filling to placement of direct restoration for comparisons of frequencies of orthograde retreatment, apical surgery and extractions, during the following 5 years.

The time interval categories were compared using Pearson’s Chi-square test.

**Results:**

No statistically significant time-dependent differences in registrations were found for orthograde retreatment (*p* = 0.089), with or without apical surgery (*p* = 0.161) and with or without extraction (*p* = 0.737).

**Conclusion:**

The time elapsed from the completion of RCT and the placement of a direct restoration did not affect the 5-year outcome of the RCT.

## Introduction

After a root canal treatment (RCT), a coronal restoration is required to rebuild the tooth for functionality and to provide a marginal seal minimizing the risk of micro-leakage. The importance of an efficient seal is supported by cross-sectional data where root filled teeth with defective marginal seals have a higher frequency of apical periodontitis [1]. Evidence is lacking for which type of coronal restoration should be advocated; either a direct restoration that can be placed during one single appointment or an indirect restoration which is manufactured outside the oral cavity, usually by a dental technician, which requires additional appointments. In contrast to many other countries, in Sweden it is most common to restore the tooth with a direct restoration following the completion of a RCT, but the timing may vary [2–4].

A temporary filling is sometimes applied after RCT pending permanent restoration. *In vitro* studies of teeth instrumented, root filled, and subsequently sealed with various types of temporary filling materials show contamination of microorganisms in the full length of the root canal after 10–14 days [5, 6]. Results from *in vitro* studies may therefore imply that the clinical outcome of RCT could be hampered by longer time between the RCT and the placement of a permanent restoration, since the temporary restoration does not provide an efficient seal [7]. The microbial leakage may in the longer perspective lead to apical periodontitis and a potential need for additional treatments such as orthograde retreatment, apical surgery or extraction of the root filled tooth. In addition, a lengthy period with a temporary restoration could conceivably increase the probability of developing cracks and fractures and jeopardizing tooth survival. Published data regarding the impact on time between RCT and crown placement are scarce but suggest that a shorter time from RCT to the crown placement is beneficial for the retention of the tooth [8, 9].

In view of the above, the time between RCT and the permanent seal may be an important prognostic factor for periapical health and thus for a future need for orthograde retreatment, apical surgery and extraction, with a short time being more favorable. Considering that more than half of world´s population has at least one root filled tooth, the knowledge has potential to improve periapical health and avoid unnecessary costs [10]. The aim of this study was therefore to investigate the possible difference in frequencies of endodontic orthograde retreatment, apical surgery and extractions of root filled teeth permanently restored with a direct restoration depending on elapsed time between completion of RCT and placement of a direct restoration. The underlying hypothesis was that the probability for failure of RCT, measured as endodontic orthograde retreatment, apical surgery, or extraction of the tooth, increases when longer periods elapse from a completed RCT until placement of a direct restoration due to microbial leakage.

### Material and methods

The study was approved by the Committee on Investigations Involving Human Subjects at Lund University, Sweden (Dnr 2011/800).

The STROBE (STrengthening the Reporting of OBservational studies in Epidemiology) checklist was used when preparing the manuscript [11].

### Study population

In Sweden, all adults are covered by the public dental care system, which provides protection against high costs for dental treatments. In 2009 all 20-year-olds or older were covered. Virtually all dentists are affiliated to the tax-funded Swedish Social Insurance Agency (SSIA). In this study, the data were retrieved from the SSIA and the data material has previously been described [3]. The data for this study included all registered orthograde RCTs on first molars in 2009. As soon as a treatment is completed, the dentist registers an item number, specific to the type of treatment. For RCTs the item numbers are 501–504 and correspond to the number of instrumented root canals in the tooth (one through four or more). For this study, only RCT first molars restored with a direct restoration (such as a composite restoration) were included, whereas teeth registered with an indirect restoration (crown, onlay or inlay) were excluded since the focus of this study was to study the effect of immediate or delayed sealing of the tooth. Item numbers for direct restorations are 701–708 depending on tooth type and extension of restoration (number of surfaces). Only one registered RCT per individual was included in the study, that being the first RCT registered in 2009 for this individual.

### Outcome

The outcome of interest for the root filled tooth restored with a direct restoration was any registration of additional endodontic treatment or extraction of the tooth, more specifically orthograde retreatment (item number 501–504), apical surgery (item number 541–542) and extraction (item number 401–404), during the follow-up period of 5 years after placement of a direct restoration succeeding RCT.

### Time interval categories

**Figure 1:**
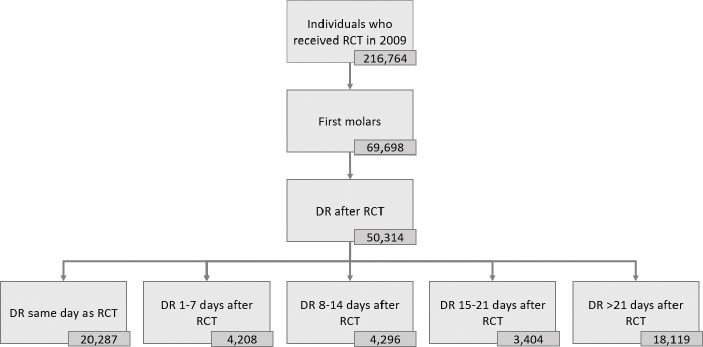
Flow chart showing the number of individuals initially registered with a root canal treatment (RCT) in 2009, after exclusion of non-first molars and unrestored/otherwise restored, and finally the number of first molars categorized in intervals according to time elapsed (0–21+ days) between completed RCT and the placement of a direct restoration (DR).

The root filled teeth were divided into five categories depending on the elapsed time between registration of the RCT during the year 2009 and registration of a direct restoration and were based on data from previously published *in vitro* studies. The five categories are seen in Figure 1; the shortest time interval was direct restoration the same day as registration of the RCT and the longest 22 days or more after RCT to reflect any problems with microleakage that could be anticipated.

Each of the three treatments indicative of an unsuccessful outcome was investigated independently in reference to the five time interval categories of direct restoration after RCT.

### Statistical analysis

Descriptive data and group comparison using Pearson’s Chi-square test were applied. *P* < 0.05 was considered statistically significant. Data were analyzed by using IBM SPSS Statistics Version 22 (SPSS Inc., Chicago, IL, USA).

## Results

According to the SSIA, 216,764 individuals in Sweden received a RCT in 2009, and approximately every 5th root filled tooth was registered with an indirect restoration. After applying inclusion and exclusion criteria, the final number analyzed were 50,314 first molars registered with a RCT and a direct restoration. The largest number of teeth (*n* = 20,287) were registered with a direct restoration on the very same day as the RCT were registered, but there were also a large number (*n* = 18,119) registered with a direct restoration after more than 21 days (Figure 1).

**Figure 2:**
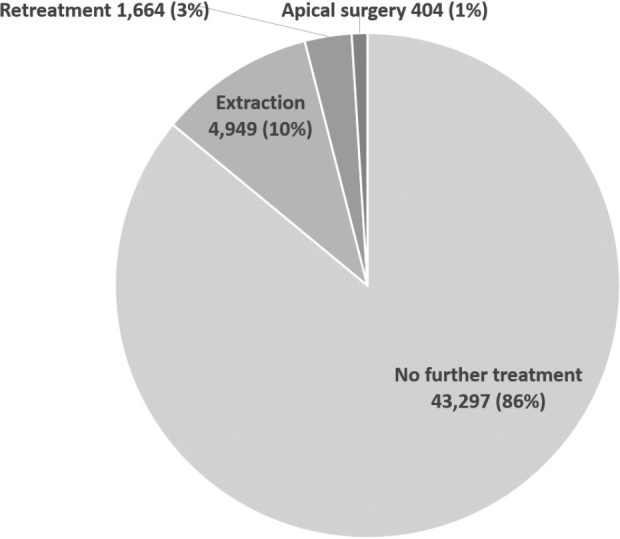
Outcome 5 years after the root canal treated molars were restored with a direct restoration; the proportion [*n*, (%)] of molars registered with orthograde retreatment, apical surgery, extraction and no further treatment.

The overall 5-year survival of root canal treated first molars restored with a direct restoration was 90%, and 86% of teeth received no further treatment during the same period. Data on the proportions of additional endodontic treatment and extractions are displayed in Figure 2 and Table 1. A large majority (85.4–86.6%) of all RCT teeth restored with a direct restoration were not registered with any additional endodontic treatment or extraction. Extractions were more frequent than orthograde retreatment and apical surgery was the most infrequent treatment. There were no statistically significant differences between the time interval categories regarding the proportion of additional endodontic treatments, that is orthograde retreatment *vs*. no orthograde retreatment (*p* = 0.089), apical surgery *vs.* no apical surgery (*p* = 0.161). There was also no statistically significant difference in the proportion of tooth survival, here presented as the proportion of extractions *vs.* no extraction (*p* = 0.737), regarding the time intervals.

**Table 1:** Proportions of additional endodontic treatments (orthograde retreatment and apical surgery), extractions and no additional treatments in each of the five time categories.

Time category*	Orthograde retreatment *n* (%)	Apical surgery *n* (%)	Extraction *n* (%)	No additional treatment *n* (%)	Total** *n*
Same day	678 (3.3)	172 (0.8)	2,056 (10.1)	17,381 (85.7)	20,287
1**–**7 days	132 (3.1)	39 (0.9)	419 (10.0)	3,618 (86.0)	4,208
8**–**14 days	173 (4.0)	37 (0.9)	418 (9.7)	3,668 (85.4)	4,296
15**–**21 days	123 (3.6)	17 (0.5)	328 (9.6)	2,936 (86.3)	3,404
> 21 days	558 (3.1)	139 (0.8)	1,728 (9.5)	15,694 (86.6)	18,119
Total ***	1,664 (3.3)	404 (0.8)	4,949 (9.8)	43,297 (86.1)	

* Elapsed time between registered root canal treatment (RCT) and direct restoration.

** Total number of teeth in each time interval category.

*** Total number of a specific treatment irrespective of time interval category.

## Discussion

Even though *in vitro* studies indicate that temporary restorations do not effectively seal the tooth following RCT and that a shorter time from RCT to the crown placement is beneficial for the retention of the tooth, the results of this study failed to show significant time-dependent difference in the proportions of nonsurgical retreatment, apical surgery or extractions in the interval 0–21+ days elapsing from completed RCT until the placement of direct restoration.

It is sometimes stated that the permanent coronal restoration providing the best prognosis for a tooth is an indirect restoration, such as a laboratory fabricated crown or only/inlay [12]. However, a Cochrane report concluded that there is currently insufficient knowledge regarding the effectiveness of indirect restorations compared to direct restorations for restoring root-filled teeth. Until more evidence is available, clinicians were recommended to rely on their clinical experience and consider the unique circumstances and preferences of their patient when selecting a restoration type [2]. The aim of this study was to investigate if the time between completed RCT and the placement of a permanent coronal restoration mattered for the outcome of the RCT, focusing on the short time periods. Direct restorations were chosen for the study since it allowed investigation of the shorter time periods that had showed potential microbial leakage *in vitro*. An indirect restoration takes multiple appointments to finish due to imprint, manufacturing and cementation of the restoration, and information regarding temporization during this process was not available from the registry. The choice to only include first molars was based on this group of teeth being the most frequently root filled, and therefore constituted the largest available sample from a single comparable tooth group [3].

The time intervals between RCT completion and direct restoration ranged from the same day to 21+ days. The intervals were selected to reflect what *in vitro* studies have reported on micro leakage; with both IRM (a polymer-reinforced zinc oxide-eugenol) and Cavit (despite layered 3.5 mm) root filled canals were infiltrated by microorganisms in less than 2 weeks [5, 6]. In addition, the use of temporary restorations increase the risk of fracture, both of the tooth and restoration, due to the temporary restorations not bonding to the tooth structure and the lack of tooth-like properties compared to resin-based composite materials [12]. The outcome measures were selected based on being core outcomes important to patient and society [13, 14]. Other outcome measures, such as healthy periapical conditions, are also important but these data were not available in this registry-based study. If such outcome measures had been available, it could potentially have shown other results; it is known that teeth with signs of periapical inflammation often remain without interventions for longer periods [15].

The data used in this study were gathered directly from the SSIA, which is a state agency and therefore considered as a reliable source; all treatments are registered due to the central insurance system in Sweden. This gives us a unique opportunity to include thousands of teeth and multiple treatments. Another advantage is that we used data already collected, and no new data were collected for the sake of this study, which is advantageous from an ethical point of view. A caveat and potential limitation is that the data from SSIA are still dependent on the caregivers’ accurate registering of treatments. Measurement errors, such as some treatments being registered on a date different from the date they were carried out, are impossible to uncover. However, the high numbers are likely to prevent occasional errors from having an impact on the overall results.

Although this study contained many teeth, there were some limitations to the certainty of the result. Since the data from the SSIA only includes registration codes referring to treatments and examinations and no patient record information or radiographs were available, no exact evaluation of a tooth’s condition can be made. It was therefore not possible to subgroup the teeth regarding diagnosis, pulp status, periapical status, and symptoms.

Contrary to our underlying hypothesis, no differences in the outcome of RCT measured as need of additional treatment were noted based on a longer time elapsed between the completion of RCT and the placement of a direct restoration. A detailed comparison with other published data was not possible since no equivalent studies could be identified. However, approximately one in 10 of the studied teeth were extracted within the 5-year follow-up; about one in about 25 teeth received retreatment and fewer than one in 100 received apical surgery, which is in line with what has been reported in other studies [16, 17]. In a study of survival of root filled teeth restored with post/core and crown, i.e. indirect restoration, the survival rate was slightly inferior when the placement of the post/core and crown was delayed more than 60 days [18]. Another study showed that root filled teeth receiving a crown more than 4 months after RCT were more likely to be extracted compared to if the crown was received within 4 months [8].

To expand the knowledge within this topic, carefully planned prospective studies are needed to avoid confounding factors. Control for the status of the teeth is necessary, both at study baseline and at follow-ups ensuring that time to permanent coronal restoration is the only between-group difference. For example, a prospective case-control design would help in determining whether a longer time elapsed from completed RCT until permanent restoration is a clinically relevant risk factor for post-treatment root canal infection. In contrast, the large number of teeth, arguably the most significant strength of this study, would not be possible in a clinical study. As so often, multiple and complementary study designs are needed to draw a complete picture of the challenge at hand.

## Conclusions

This large registry-based study aimed to investigate whether immediate or delayed placement of a direct restoration after completed RCT affected the outcome of the RCT, measured as performed additional treatments, including extraction. In this 5-year follow-up, no such differences were identified. Prospective controlled clinical studies are needed to control for clinical or tooth-related factors that may confound the outcome.
